# Remanufactured circular mapping catheters: safety, effectiveness and cost

**DOI:** 10.1007/s10840-018-0497-x

**Published:** 2018-12-26

**Authors:** Lisa WM Leung, Banu Evranos, Alexander Grimster, Anthony Li, Mark Norman, Abhay Bajpai, Zia Zuberi, Manav Sohal, Mark M. Gallagher

**Affiliations:** Cardiology Clinical Academic Group, St. George’s University Hospitals NHS Foundation Trust, St. George’s, University of London, St George’s Hospital, Blackshaw Road, London, SW17 0QT UK

**Keywords:** Remanufacturing, Cardiac electrophysiology catheters, Circular mapping catheters, Infection risk, Sterilisation, Safety, Cost-effectiveness

## Abstract

**Background:**

The use of remanufactured single-use devices (SUDs), including cardiac electrophysiology catheters, has become established in the USA and other health care systems but without much published scientific evaluation on the relative safety or efficacy of these devices. In the United Kingdom (UK), the use of remanufactured SUDs has not been routine. We performed a structured evaluation of the safety and efficacy of a remanufactured circular mapping catheter (Stryker® remanufactured Lasso NAV 2515) during its introduction in our centre.

**Methods:**

We prospectively evaluated the performance of a remanufactured circular mapping catheter in 100 consecutive patients undergoing an AF ablation. Operator feedback was obtained, assessing the device appearance, ease of use and function. As an indirect measurement of efficacy, acute procedure metrics were compared to those in 100 propensity-matched cases performed by the same operators using a new device. Cost savings were calculated.

**Results:**

No complication occurred in association with the remanufactured device. There was one reported failure of device malfunction—the flexion-extension mechanism of a remanufactured catheter and none in the matched-control group. There was satisfactory communication with the electro-anatomic mapping system. Ease of use of the remanufactured catheter was reported to be similar to a newly manufactured device. Procedural duration was similar with remanufactured devices and matched controls. With 100 cases using the remanufactured device, cost savings amounted to £30,444.

**Conclusions:**

The use of remanufactured circular mapping catheters is safe, efficient and reliable. Widespread use of remanufactured SUDs offers the possibility of significant economic benefit.

## Background

The use of remanufactured single-use devices (SUDs), including electrophysiology (EP) catheters, has been established for decades in the USA. [1] Re-use of catheters was widespread in the early years of electrophysiology, but this generally involved just rudimentary checking, cleaning and re-sterilisation of the catheter within the healthcare institution, a practice that declined in response to the labelling of devices as ‘single-use only’. Modern remanufacturing is performed on an industrial scale with rigorous adherence to defined protocols, producing a remanufactured device which has been performance-tested and shown to meet the original equipment manufacturer (OEM) requirements. The verifiable elimination of infective agents is central to the process.

The financial burden on healthcare systems is ever increasing and the cost of running a cardiac EP laboratory is high. The prevalence of atrial fibrillation in Europe is estimated to be over 6 million, and this is likely to increase over the oncoming decades based on recent population studies [2]; a significant proportion of these patients will go on to require catheter ablation including repeat procedures. The incentive for investigating the integration of remanufactured cardiac EP catheters as part of routine use is to provide a sustainable approach to cost reduction in the cardiac EP laboratory.

The remanufactured device is treated as a completely different product in the device industry; the responsibilities remain specific to the remanufacturing company and remanufacturing industry regulations, completely separate to the original device and the manufacturer. Despite this and the fact that remanufacturing has the capacity to alter the characteristics of a device, no remanufactured SUD has been the subject of a structured evaluation published in the scientific literature. Over the many years of widespread use of remanufactured SUDs in everyday practice in the USA, there have been no major adverse events attributable to the remanufacturing process; conversely, there is little published scientific evidence to confirm the safety of this approach and none specific to EP catheters [1, 3–7].

**Fig. 1:**
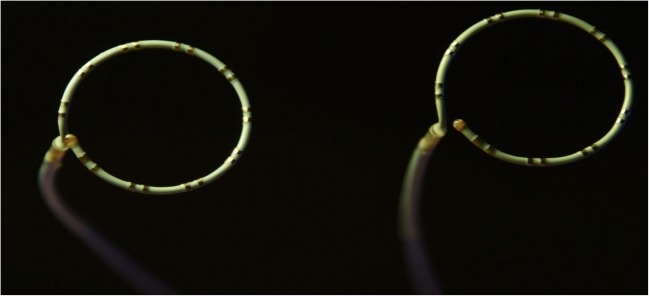
Photograph of the Stryker® remanufactured Lasso NAV 2515 circular mapping catheter (left) and the original device, Lasso® (right). Other than the markings on the handle of the device, we have not been able to identify any difference on careful inspection of each remanufactured device compared to the newly manufactured version

The Lasso® mapping catheter (Carto® Lasso® 2515 Eco, Biosense-Webster® Johnson&Johnson, Diamond Bar, USA) is a specialised device with a tip that is fashioned into a circular geometry (Fig. 1). The circular component can be adjusted in its diameter. The shaft can be flexed or extended. In a typical procedure, the Lasso® catheter is expected to be adjusted frequently and exposed to torque and linear stress, cardiac movement and other catheter movement for a duration of over 2 h. The purpose of this study was to prospectively evaluate the safety and efficacy of a remanufactured version of the Lasso® 2515 eco Variable Catheter during AF ablation procedures compared to propensity matched control procedures performed using the newly manufactured version of the catheter.

## Methods

We prospectively studied 100 consecutive patients undergoing elective AF ablation using a remanufactured circular mapping catheter (Stryker®, Michigan, USA), a remanufactured version of the 22-pole model of the Lasso® 2515 eco Variable Catheter (Biosense-Webster®), with an electro-anatomic system (Carto®, Biosense-Webster®). All patients were propensity-matched to cases performed using a previously unused circular mapping catheter of the same model (the ‘first-use’ group) selected from a database of 806 AF ablations performed over the previous 4 years.

A record was made of any catheter-associated problem that occurred, and a structured interview was held with the operator immediately after the procedure to evaluate the performance of the catheter. After the procedure, the catheter was inspected for any defect in the electrodes or the insulation, for any deformation of its curve, or any malfunction in its deflection mechanism.

The parameters assessed were catheter-related complications during or after the procedure, ease of handling the catheter during the case, failure of electrodes to record electrograms or to stimulate appropriately, failure of communication with the electro-anatomic mapping system, and physical defect or deformation of the catheter on inspection after use. Indirect markers of catheter function were also measured including procedure duration and fluoroscopy duration. Complications that did not have any likely relationship to the catheter were also recorded up until the point of discharge from hospital, including any major adverse cardiovascular/cerebrovascular events (MACCE), vascular injury, or cardiac tamponade.

Patients were followed up at 3 months after the procedure. Medical records were reviewed for evidence of complications of the procedure occurring in the period within 3 months after ablation, and for any pyrexial or infective illness reported in this period. Total cost savings to the department was calculated.

### Statistics

To estimate the propensity score, we used logistic regression including the following co-variates: age, gender, performing operator and procedural complexity characteristics. Matching was performed with the nearest neighbour method using a 1:1 ratio (R extension pack - R version 2.15.0). Analysis was conducted between the matched cohorts. The level of significance was set to 0.05. Analysis was performed with IBM SPSS statistical software (version 22.0; IBM SPSS Statistics, NY, USA).

## Results

**Fig. 2:**
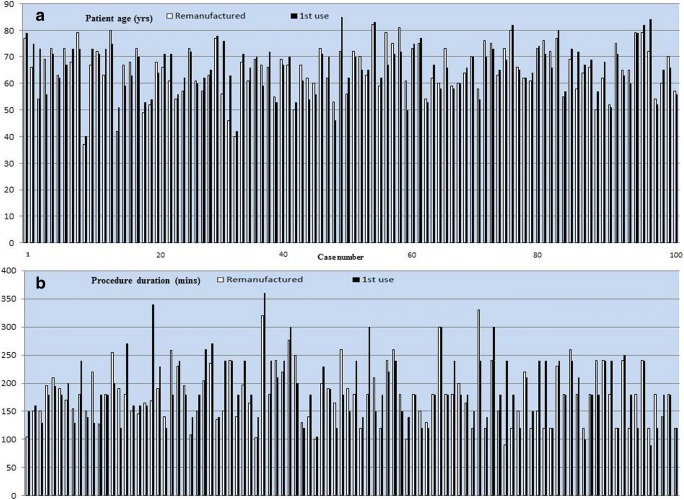
Comparative graphs of remanufactured and first-use circular mapping catheters. **a** Patient age (years). **b** Procedure duration (min). Graph a is to demonstrate adequate propensity matching, which apart from age also includes patient gender and operator. Graph b shows no significant overall difference in procedure times in the study or matched controls

The study patients (remanufactured) and the control (first use) population were well matched for demographic and procedural characteristics (Fig. 2; Table 1). All of the Stryker® remanufactured catheters used were at the first reuse cycle (the NAV version can only be reused once). No complication occurred in either group that could be attributed to the performance of the circular mapping catheter. Of other complications, there was one case of cardiac tamponade in the matched-control group.

**Table 1:** Patient characteristics

Parameters	Remanufactured	First use
Male (*n*)	68	68
Female (*n*)	32	32
Overall average age	67.1± 8.5 SD	65.8± 9.1 SD
AF subtype (*n*)		
PAF	37	37
Pe-AF	33	33
Redo cases	30	30

There was only one confirmed case of failure of a remanufactured catheter. The flexion-extension mechanism completely failed mid-procedure, having performed normally until that point. The catheter was removed without difficulty and the procedure completed with a replacement catheter. Assessment of the catheter confirmed the failure of the flexion mechanism due to the contraction puller wire coming off the ferrule. In one other case, the operator returned a remanufactured catheter for retesting after the procedure due to a perceived limitation in flexion, but on retesting against OEM standards, the device was found to function normally. There was no negative feedback about the mechanical function of the remanufactured catheter in other cases. There were no instances of physical defects or deformation of the catheter upon inspection after use. The quality of electrograms and the communication between the catheter and the electro-anatomic mapping system and stimulator were satisfactory in all cases. There was no report of sub-optimal performance among the 100 matched control cases.

The procedure duration was similar in the study group and matched controls (‘remanufactured’ 178.9 ± 51.3 versus first use 189.5 ± 55.3, *P* = 0.16) (Table 2). The first-use group had higher overall fluoroscopy duration compared to the remanufactured group (21.5 ± 13.5 versus 11.8 ± 9.6 min, respectively, *P* < 0.0001).

**Table 2:** Procedural characteristics

Parameters	Remanufactured	First use	*P* value
Procedure duration (min)			
(a) PAF	157.9 ± 45.7 SD	164.2 ± 44.7 SD	*P* = 0.55
(b) Pe-AF	208.5 ± 49.1 SD	226.8 ± 59.3 SD	*P* = 0.18
(c) Redo cases	172.4 ± 46 SD	179.7 ± 39.6 SD	*P* = 0.51
Fluoroscopy duration (min)	11.8 ± 9.60 SD	21.5 ± 13.5 SD	*P* < 0.0001
PVI only (*n*)	37	37	N/A
PVI+ (*n*)	51	51	N/A
Other (CFAE, linear) (*n*)	12	12	N/A
Patient major complication (*n*)	0	0	N/A
Patient minor complication (*n*)	0	0	N/A
Mapping catheter failure (*n*)	1	0	N/A
Other catheter failure (*n*)	0	0	N/A

Comparing the list prices, the remanufactured circular mapping catheter was 45% cheaper than the newly manufactured version. The cost savings to our department arising from these 100 cases amounted to £30,444.

## Discussion

St. George’s Hospital is the first UK centre to have ‘real-world’ experience on remanufactured electrophysiology catheters. We have tested the use of the remanufactured Lasso NAV 2515 circular mapping catheters prospectively and found it to be as safe and as efficient as a newly manufactured device. There was no acute complication among the 100 cases studied. This study supports the integration of remanufacturing into UK and EU healthcare systems as a sustainable approach to cost-effectiveness that also maintains safety and efficacy standards for our patients.

### Mechanical performance

A deformed or broken circular mapping catheter could be a dangerous instrument. Deformation of the circular geometry could cause the tip to protrude and potentially lacerate the wall of the left atrium. Deformation of the shaft could increase the risk of catheter entrapment in the mitral apparatus; defects in the insulation of the catheter could provide a stimulus to thrombosis and increase the risk of systemic embolisation. Any issues associated with catheter malfunction would be expected to manifest during or immediately after the procedure, or would be revealed by inspection of the catheter at the end of the procedure. The absence of major problems observed therefore assures us that the remanufacturing process is robust in limiting the potential for mechanical defects.

The observed 1% catheter failure rate among the remanufactured catheters was not replicated in the matched control cases but is in keeping with the long-term experience of our unit. We have recorded five instances of confirmed failure of first-use circular mapping catheters over 5 years, during which period over 800 such catheters were used. There were a range of faults including damaged connections, puller wire coming off the ferrule and internal failure of the sensor.

Procedure duration times were similar in the remanufactured and first-use groups, supporting the impression that the remanufactured catheters functioned to the standard expected of an original device. The catheters were used in a wide mix of AF ablation cases, ranging from PVI to more complex and lengthy procedures, some lasting over 4 h. Only fluoroscopy times differed between the remanufactured and first-use groups. The control cases were from a historic database, and we believe that this difference reflects the progressive decline in reliance on fluoroscopy seen in our unit in recent years.

### Validating mechanical performance of remanufactured SUDs

**Fig. 3:**
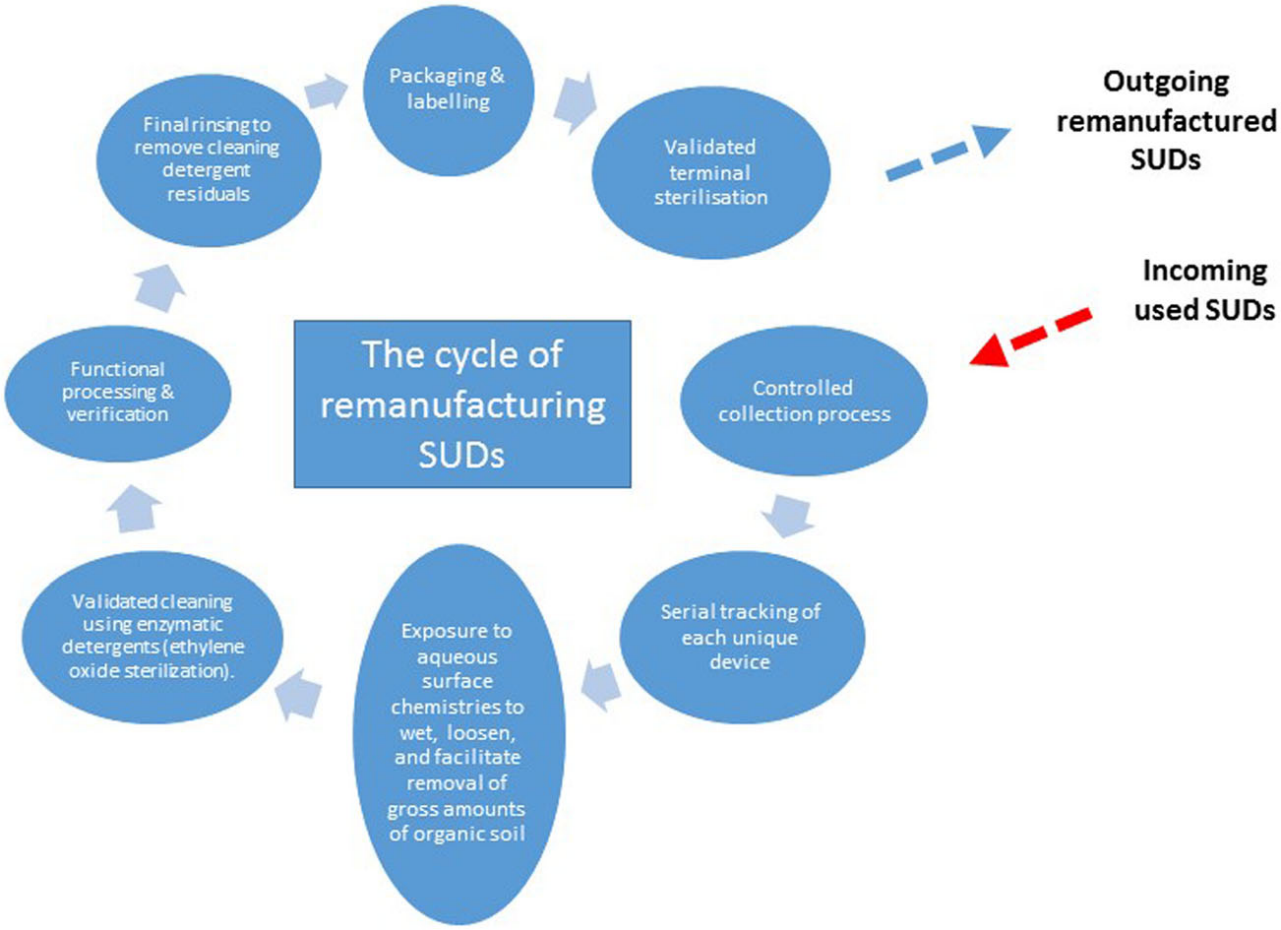
The cycle of remanufacturing single-use devices (SUDs)

**Fig. 4:**
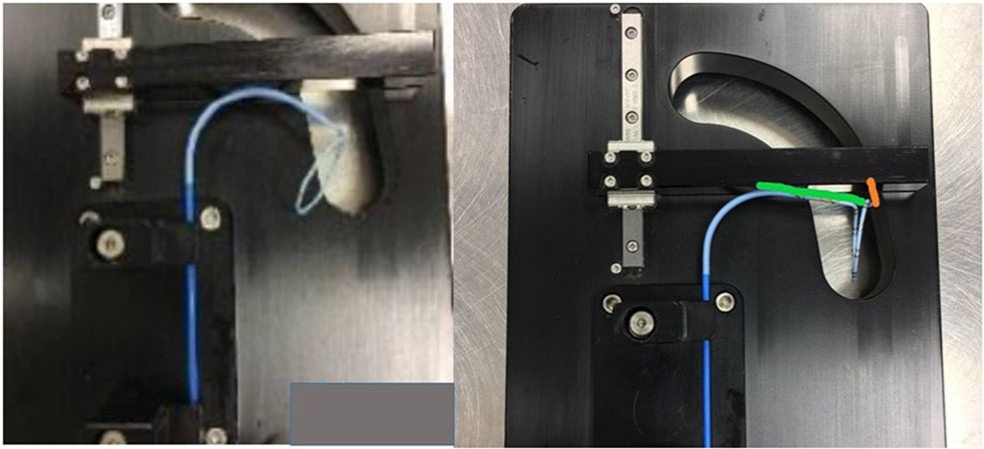
Original equipment manufacturer (OEM) tests against a template. The “*D* curve” of the circular mapping catheter is tested; the annotated green line measures the angle created by the shaft of the catheter. The orange line represents the angle of deflection from the catheter tip and has to be greater than 0 to pass the test

The remanufactured catheters studied in this series had all undergone a controlled and structured approach in the cycle of remanufacturing (Fig. 3). All catheters were tested against OEM standards on entering the remanufacturing cycle. The testing involved a number of redundant quality control steps testing torsion, deflection, joint seal integrity and tensile strength to verify manoeuvrability, stability and reliability. Curves were measured against standard templates to assess the direction, shape and plane of the curve against predetermined criteria. The circular mapping catheter had to flex to a ‘*D* curve’ (Fig. 4). Over- or under-flexion or deflection from the plane of flexion would not have met OEM standards and would therefore have been deemed unacceptable. The tests were completed three times for each catheter. Each remanufactured device has a unique code on the device handle and on the packaging to track its progress through the system, and if appropriate, the device details can be traced back to its origin and to determine its reuse cycle (first reuse or second reuse). This will also enable any device-related issues to be fed back to the company.

### Infective agents

The reuse of medical devices and surgical equipment carries a small but important risk of transmission of infective agents. Prions are a particular concern in the UK, which was the epicentre of the outbreak of variant Creutzfeldt Jakob disease from 1996 onwards and still accounts for a large majority of cases worldwide [8]. The medical literature includes more than 400 reported cases of iatrogenic Creutzfeldt Jakob disease transmission, mostly through the use of pituitary extracts and dura mater grafts, but also from neurosurgical instruments and blood transfusions [9]. Prions are resistant to proteolytic enzymes and remain pathogenic even after long periods of time and exposure to high heat of up to 200° [10].

Viruses are also of concern, particularly the widespread hepatitis C virus (HCV). Iatrogenic hepatitis C transmission is not common, and when it occurs is usually from a HCV-infected operator [11]. Druce et al. performed experimental studies assessing the efficacy of various sterilisation protocols in eradicating hepatitis and Coxsackie viruses from used cardiac electrophysiology catheters [12]. They found that blood borne viruses could be eliminated by exposure to ethylene oxide, an alkylating chemical which effectively inactivates DNA of microorganisms; virus nucleic acid persisted in catheters that had been simply washed in detergent or exposed to enzyme cleaners.

The remanufacturing of the catheters involved in the current study included a validated protocol to eliminate infective agents, involving thorough cleaning and exposure to the ethylene oxide. Ethylene oxide has been shown to be compatible with the polyurethane that forms most of the external surface of the catheter, and does not affect its appearance or physical characteristics. Other forms of sterilisation may induce a brittle or ‘yellowing’ quality to plastic polymers and can create additional friction between interacting catheter surfaces. Although no instance of transmission of infection by an electrophysiological catheter has been reported, it must be noted that it is notoriously difficult to fully assess prion transmission as prion-related conditions may remain indolent for decades.

### Regulations governing remanufacturing of SUDs

In the USA, the Food and Drug Administration (FDA) has reviewed and officially reported on the remanufacturing industry. They have found no evidence that the use of remanufactured devices increased the risk to health, with over two decades of experience [1]. However, there is a lack of peer-reviewed studies into the remanufactured devices and so we are still without comprehensive assessment into the relative safety of these devices. FDA data-collection methodology has recently become more comprehensive, and this will probably yield clearer information on the performance and safety of reprocessed devices in the future.

The Medicines and Healthcare products Regulatory Agency (MHRA) permits the practice of remanufacturing SUDs within strict guidelines [13–16]. There should be adherence to the medical devices directive and a CE mark for each product. The remanufacturing company must transfer the devices in a closed loop system between themselves and the healthcare provider. Guidelines are in place to monitor decontamination and sterilisation processes, as well as adverse event reporting via the European Union (EU) Medical Devices Vigilance System [17, 18].

### Other reprocessed medical devices

Other cardiac electrophysiology catheters are available as remanufactured versions, including decapolar coronary sinus catheters, but the same processes could be applied to many other models. We began our experience of remanufactured catheters with the circular mapping catheter as it is particularly intricate in its construction and is particularly subject to stress during routine use. If this catheter can withstand the remanufacturing process in good condition and survive a second procedure, we believe that other catheters are likely to perform at least equally well. Other medical devices available in the remanufactured form include devices used in general and laparoscopic surgery, for example endoscopic trocars and balloon inflation devices.

### Cost implications

Based on list prices, we have calculated the cost savings to our department arising from these 100 cases at £30,444. An estimated 5000–10,000 ablations requiring the use of a circular mapping catheter are performed annually in the UK [19, 20], implying the potential for an annual saving of £1,700,000 if half of these were switched to the remanufactured version. The overall potential cost savings from all reprocessed SUDs is estimated at £17 million per annum in the UK alone with scope to increase [17]. In 2017, the total cost savings to the US healthcare system from the remanufacturing of SUDs was estimated to exceed $326,000,000 [21]. Published savings from individual centres were also significant: WakeMed and Duke were able to save $750,000 and $839,000, respectively, in the years 2013–2014.

Integration of remanufactured devices into everyday clinical practice would be a sustainable cost reduction strategy to the cardiac EP department; the safety of this practice should be accounted for by careful external regulation alongside regular internal auditing by each individual department. This cost reduction method may enable continued expansion of AF ablation services and so meet the ever increasing demand for catheter ablation treatment.

The cost benefit arising from the remanufacturing of SUDs is of even greater importance in less wealthy nations. AF is a world-wide problem, but AF ablation is disproportionately available to richer populations; others must endure the disability of the arrhythmia or the dangers associated with antiarrhythmic medications. Reducing the cost per procedure should permit the treatment to more of the population in need.

## Conclusion

The remanufactured circular mapping catheter proved to be safe and efficient with no adverse events and with satisfactory catheter performance. The incorporation of remanufactured SUDs into standard practice in a carefully regulated system could provide significant economic benefit without compromising safety.

## Limitations

Because of the relatively small study population and short duration of follow-up, our study cannot exclude the possibility of transmission of infective agents; further study over a longer duration would be required to establish absolute safety from transmission of infection. The study was not ‘blinded’ so operator bias cannot be excluded.
